# Evaluating the feasibility and impact of case rate payment for recovery support navigator services: a mixed methods study

**DOI:** 10.1186/s12913-020-05861-8

**Published:** 2020-11-03

**Authors:** Maria E. Torres, Mary Brolin, Lee Panas, Grant Ritter, Dominic Hodgkin, Margaret Lee, Elizabeth Merrick, Constance Horgan, Jonna C. Hopwood, Andrea Gewirtz, Natasha De Marco, Nancy Lane

**Affiliations:** 1 Brandeis University, Heller School for Social Policy and Management, Waltham, MA 02453 USA; 2 Smith College School for Social Work, Lilly Hall, Northampton, MA 01060 USA; 3 Massachusetts Behavioral Health Partnership, a Beacon Health Options company, 1000 Washington Street, Suite 310, Boston, MA 02118 USA; 4 Harvard Medical School and Harvard Pilgrim Health Care Institute, 401 Park Drive, Suite 401 East, Boston, MA 02215 USA

**Keywords:** Substance addiction, Patient navigation, Third-party payers, Mental health recovery, Patient engagement, Payment reform, Case rate, Detoxification, Recovery support

## Abstract

**Background:**

Acute 24-h detoxification services (detox) are necessary but insufficient for many individuals working towards long-term recovery from opiate, alcohol or other drug addiction. Longer engagement in substance use disorder (SUD) treatment can lead to better health outcomes and reductions in overall healthcare costs. Connecting individuals with post-detox SUD treatment and supportive services is a vital next step. Toward this end, the Massachusetts Medicaid program reimburses Community Support Program staff (CSPs) to facilitate these connections. CSP support services are typically paid on a units-of-service basis. As part of a larger study testing health care innovations, one large Medicaid insurer developed a new cadre of workers, called Recovery Support Navigators (RSNs). RSNs performed similar tasks to CSPs but received more extensive training and coaching and were paid an experimental case rate (a flat negotiated reimbursement). This sub-study evaluates the feasibility and impact of case rate payments for RSN services as compared to CSP services paid fee-for-service.

**Methods:**

We analyzed claims data and RSN service data for a segment of the Massachusetts Medicaid population who had more than one detox admission in the last year and also engaged in post-discharge CSP or RSN services. Qualitative data from key informant interviews and Learning Collaboratives with CSPs and RSNs supplemented the findings.

**Results:**

Clients receiving RSN services under the case rate utilized the service significantly longer than clients receiving CSP services under unit-based billing. This resulted in a lower average cost per member per month for RSN clients. However, when calculating total SUD treatment costs per member, RSN client costs were 50% higher than CSP client costs. Provider organizations employing RSNs successfully implemented case rate billing. Benefits included allowing time for outreach efforts and training and coaching, activities not paid under the unit-based system. Yet, RSNs identified staffing and larger systems level challenges to consider when using a case rate payment model.

**Conclusions:**

Addiction is a chronic disease that requires long-term investments. Case rate billing offers a promising option for payers and providers as it promotes continued engagement with service providers. To fully realize the benefits of case rate billing, however, larger systems level changes are needed.

**Supplementary Information:**

The online version contains supplementary material available at 10.1186/s12913-020-05861-8.

## Background

A high percentage of people with substance use disorders (SUD) experience more than one detoxification (detox) admission within a 12-month period [1–5]. Detox services at freestanding detox centers in Massachusetts provide medically monitored inpatient detoxification. Massachusetts data on detox admissions from 1996 to 2002 found individuals using detox services averaged five detox admissions a year [3]. More recent data from the Massachusetts Behavioral Health Partnership (MBHP), a Beacon Health Options company which manages behavioral health for many Massachusetts Medicaid enrollees, found that 57.7% of those receiving detox services in FY2011 had two or more admissions that year, and that this group of “repeaters” accounted for 87% of all detox admissions that year [6]. Repeated admissions to detox without engagement in follow-up care represent lost opportunities for recovery, highlight inadequate access to a limited resource, and come at significant human and financial costs [5, 7, 8].

Successfully navigating the transition from detox to SUD treatment is crucial. Yet, continuity of care studies indicate that in most settings less than half of clients utilize post-detox SUD treatment within 14 days of discharge [9]. Various states fund services to support post-detox linkage. For example, Massachusetts offers Medicaid-billable community support program (CSP) services upon discharge from a detox. CSP offers support services to help consumers at high risk of relapse to access and use community-based behavioral health services [10]. Through their employer, CSP workers receive annual training on such topics as engagement and outreach, service coordination and principles of recovery; each employer determines which trainings will be offered [10]. CSP services are billed in 15-min units of service and require prior authorization from the insurer. Additionally, clients must meet medical necessity criteria. Strict criteria detail the range of services CSP workers can provide, which includes in-person or telephone case management, direct time with clients and providers, and travel, but not outreach services [11].

Despite patients’ access to CSPs, data from MBHP indicates that only 30% of eligible clients see a CSP upon discharge from detox. Key factors that may contribute to low engagement include issues with the payment model and staffing. SUD treatment typically pays fee-for-service (FFS), which has both strengths and weaknesses. FFS payments incentivize more care and provide higher payments for clients needing more care [7, 12, 13]. FFS payments do not reward quality care and restrict the scope of services covered (e.g. outreach not covered) [12]. Given the complex needs of high-frequency users of detox services, these drawbacks are significant.

Case rate payment, may offer a better option than FFS payment to reimburse care navigator services for patients with repeated detox admissions. Under a case rate payment, providers receive a previously negotiated flat-rate for services delivered over a certain period [14]. Case rate payment offers a cost-effective way to pay for services for individuals with complex needs [12, 13, 15]. A case rate can allow RSN workers to have greater flexibility in their work with clients, allowing more outreach and follow up and, with no penalty to their income, they might participate in more training and professional development, improving quality of care. Moreover, a case rate system reduces administrative burden for both providers and payers as it does not require the same level of tracking and billing infrastructure.

Case rate billing has not been tested for RSN or CSP services among those with repeated admissions to detox. As a component of a larger study, this sub-study addressed this gap, assessing the feasibility and impact of case rate payment for RSN services using the Medical Research Council (MRC) framework for the evaluation of complex interventions [16]. This framework, provides guidance on process evaluation of interventions that contain many interacting components, particularly when the key aim of the evaluation is to determine whether it is effective in everyday practice [17]. As such, the MRC framework focuses on assessing the overall implementation and impact of interventions taking into consideration contextual factors [16]. The overarching research question that guided this study asked: *Is case-rate payment for RSN services a feasible alternative to unit-of-service billing currently in place for CSP services for clients with repeated admissions to detox within a 12-month period?* Feasibility can be determined using many different criteria including effects on subsequent detox admissions or overall health care costs. Those criteria are described elsewhere [18]. For this component of the study, feasibility was determined based on the following criteria: (1) Did case rate billing provide an economical alternative to FFS billing for this population? (2) Did the case rate payment method improve client length of engagement for clients receiving RSN services compared to clients receiving CSP services paid FFS? and (3) How did advantages and disadvantages of the case rate payment method influence provider-level practices?

## Methods

### Study setting

This mixed methods study took place from March 2013 through March 2015, under a grant from the Centers for Medicare and Medicaid Services (CMS) to MBHP, which manages behavioral health services for a portion of the MassHealth (Massachusetts Medicaid) Program. Brandeis University was MBHP’s academic partner. All detox providers funded by MBHP were invited to participate in the study. Site visits to describe the study and address any questions about participation were made to all invited programs. There was no consequence for non-participation. Of the 14 provider organizations invited, 13 choose to participate. The one provider that declined the invitation to participate did not provide a reason for their decision. This provider served the same population as participating programs and was not substantively different from participating programs in terms of location, criteria for admission, or services provided. Detox providers willing to join the study were assigned to either Treatment-As-Usual (TAU, nine sites) or Intervention (four sites) group based on client volume. Our aim was to minimize the number of providers offering the intervention to allow the research team to closely monitor implementation. Data from the year before the study (FY2011) indicated that the four intervention providers covered 54% of the detox units of service that year [6]. Providers in both groups represented urban and suburban settings and larger and smaller programs.

Within the study, RSNs performed similar tasks to CSPs but received more extensive training and coaching and were paid via the experimental case rate. TAU detox programs continued to employ CSP workers. At intervention detox sites, CSP workers were trained to become Recovery Support Navigators (RSNs). As needed, intervention sites also hired new staff who were trained as RSNs. RSNs provided CSP-like services with a few notable differences. All RSNs completed an initial training and participated in monthly in-person coaching sessions and quarterly trainings as part of a larger study, described elsewhere [19]. Organizations providing RSN services were paid a daily case rate for each client on RSNs’ caseloads, and RSNs had no restrictions on the type or volume of services they provided to clients. However, RSNs needed to meet/connect with each client at least once within a 30-day period to qualify for continued payment. To capture the type and volume of services provided, RSNs tracked the nature, type and frequency of each client contact via a weekly RSN service log.

### Data sources

Quantitative data came from MBHP Medicaid claims data and RSN service data for RSN clients. For CSP clients, the only service delivery data captured were CSPs’ requests for service authorization.

Qualitative data were collected by the two lead authors and a graduate research assistant, who are trained in qualitative methods, using key informant interviews and detailed meeting notes from RSN and CSP Learning Collaboratives (LCs). Research staff conducted ten one-hour in-person interviews with RSN staff at each of the four intervention sites during the second year of the study to develop a more in-depth understanding of the training and support needed by RSNs, the benefits and challenges of offering RSN services and the impact of other aspects of the intervention on their work. Seven interviews with RSNs and three interviews with RSN supervisors were completed. The interviewers did not have relationships with the provider staff prior to the study, but at the study’s outset they shared the purpose and goals of the study with provider staff. The qualitative research team invited the RSN supervisors and key RSN staff, those whose caseloads were predominantly RSN cases, to take part in the interviews. In total, only one RSN supervisor was not available for the interview. All others agreed to take part in the interview. Interviewees signed a consent form prior to the interview. There was no financial compensation for those who chose to participate. Interviewers used an interview guide, took notes and requested permission to audio record the interviews. The research team wrote detailed notes from the audio recordings and coded them with their field notes to summarize and identify themes. LCs were held at MBHP’s offices when in-person (10 meetings) and by telephone (4 meetings) and conducted in the second and third years of the study. LC participants included representatives from the evaluation team and key staff from participating RSN and CSP providers. Fourteen 90-min LCs were held, seven for intervention providers (11 attendees) and seven for TAU providers (12 attendees). Themes discussed in the LC included questions about barriers and facilitators for clients who had access to RSN or CSP services, service delivery challenges, as well as questions about other aspects of RSN and CSP service delivery. The meetings had formal agendas developed by the research staff, who took detailed notes and wrote individual meeting summaries and annual summaries. One to three representatives from MBHP were sometimes present at the LC to answer study related questions, present preliminary claims data, or address RSN or CSP service delivery questions or concerns. The key informant interviews and LCs offered opportunities to learn about implementation successes and challenges.

### Participant eligibility criteria and recruitment

Study participants were members of the MassHealth Primary Care Clinician (PCC) plan aged 18–64 whose behavioral health care was managed by MBHP. Plan members became study eligible if (1) they were admitted to a detox program and had received authorization for a detox admission at least once in the prior 12-month period, (2) this “index admission” took place between March 29, 2013 and March 31, 2015, and (3) they lived within the respective TAU or intervention provider catchment area. Members choosing to receive services were classified between the RSN and CSP analytic samples based on the location of their index admission (intervention or TAU). The study protocol was reviewed and approved by the New England Institutional Review Board.

### Study measures

Study variables included client gender, age, whether there was any prior detox within 90 days, Charlson Comorbidity Score [20], mental health diagnosis, Medicaid enrollment category, and length of Medicaid enrollment. ICD-9 codes in the claims data were used to determine whether a comorbid mental health diagnosis was present, and to construct the Charlson Comorbidity Score for number of chronic conditions. This index is computed by counting how many comorbid illnesses a patient has from a pre-established list of 17, and assigning weights greater than 1 to certain more severe illnesses [20].

CSP claims data were analyzed at both the person level and the service unit level (15-min units), while RSN data were analyzed at the person level and the case rate level (a daily rate paid in monthly increments). Costs were reported on a *per member* and *per member per month* basis, to adjust for potential differences between case rate and unit level billing.

To determine total costs per member over the entire study, the number of units billed for CSP services and the number of days billed for RSN services post-index detox admission were identified, and multiplied by the corresponding payment rates. Per member per month costs were calculated using the number of units or days billed in the month multiplied by the corresponding payment rate. Length of engagement was calculated as days between the first and the last service day. Claims data included 816 RSN clients and 924 CSP clients. RSN service data included 799 clients; 17 clients were missing RSN service data.

### Analytic approach

Descriptive statistics assessed differences between the larger study sample and the subset of clients who engaged in RSN or CSP services. For the larger study sample, eligible clients were assigned to a study arm based on intention-to-treat. Client assignment depended on the treatment program they were admitted to when they became eligible for the study, their “index” admission. Assignment was fixed for the length of the study, and was not dependent on the client’s decision to accept RSN or CSP services. The intention-to-treat approach is less vulnerable to selection effects which could bias the analyses if they had been limited to only those subjects who accepted services. To answer the research questions, greater emphasis was placed on the quantitative data, using the qualitative data to provide context and nuance to the findings. Feasibility of the case rate intervention was assessed by comparing per member per month and total costs between CSP and RSN clients. Emphasis was given to the per member per month cost, underscoring the importance of longer engagement with services.

To determine how the case rate impacted service delivery, analyses focused on the type of contact and amount of time spent on each task. CSP staff cannot bill for outreach attempts, thus all CSP contact is defined as service contact. RSN service data (*n* = 799) differentiated the type of contact (outreach or service contact) and noted the amount of time spent on each task.

Qualitative data on the role and impact of provider-level context were coded by the three qualitative interviewers, with themes derived from the data, to summarize, synthesize and sort the information for analysis. All data were double coded and differences between coders resolved. The resulting coding tree included three themes with seven categories or parent codes across the themes and twelve child codes across the categories. Framework analysis was used to identify key themes and to provide context to the quantitative analyses. This method has been used with increasing frequency in health services research to provide a structured means for reducing qualitative data while maintaining the context of data within and across interviews; it is both inductive and deductive in its thematic analysis and facilitates comparison across cases [21–23]. Results were shared with the LC to get their feedback and clarification.

## Results

Table 1 presents basic characteristics of the full study sample alongside data about the subset of clients who utilized RSN or CSP services. Thirty-one percent of intervention site clients utilized RSN services during the study, while 51% of TAU clients utilized CSP. Focusing on those that utilized support services, the data indicates significant differences between RSN and CSP clients. The RSN sample included proportionately more females, and had more clients ages 18 to 24 and fewer clients aged 40 and older. RSN clients were less likely to have had a detox admission within the past 90 days, had fewer comorbid conditions and were less likely to have disability as their Medicaid enrollment category.

**Table 1:** Characteristics of Larger Study Sample Alongside Characteristics of Clients Who Utilized RSN or CSP Services

	Full Claims Data Sample (***N*** = 4491)			Subset of clients who utilized RSN or CSP Services (***N*** = 1740)		
	Intervention	TAU	***p-value***	RSN clients	CSP clients	***p-value***
Number of clients	2667 (59.4%)	1824 (40.6%)		816 (46.9%)	924 (53.1%)	
Gender^a^			0.00			0.05
Female	34.8%	28.6%		35.5%	31.1%	
Male	65.2%	71.4%		64.5%	68.9%	
Age			0.00			0.00
18–24	13.7%	8.4%		14.6%	7.7%	
25–29	20.5%	15.8%		18.4%	16.5%	
30–39	30.2%	30.3%		30.5%	31.2%	
40+	35.6%	45.5%		36.5%	44.6%	
Prior detox past year						
≤ 90 days	51.6%	53.0%	0.35	51.5%	56.4%	0.03
Charlson Comorbidity Score (0 = no comorbidity; higher score indicates more comorbidity)			0.00			0.00
0	59.1%	51.5%		60.7%	48.6%	
1	25.0%	25.7%		24.5%	27.8%	
2	7.9%	9.9%		7.6%	11.0%	
3	2.6%	3.7%		2.2%	3.6%	
4+	5.4%	9.2%		5.0%	9.0%	
At least 1 Mental health diagnosis	77.8%	79.0%	0.34	79.5%	83.1%	0.06
Medicaid Enrollment Category			0.00			0.00
Disabled	29.0%	38.1%		35.3%	42.4%	
Non-disabled (TANF)	22.7%	17.5%		29.6%	16.7%	
Basic	8.3%	10.0%		7.8%	9.7%	
Essential	40.0%	34.4%		27.3%	31.4%	
Medicaid enrolled prior to index detox			0.53			0.40
≤ 1 year	18.9%	18.1%		16.5%	18.0%	
> 1 year	81.1%	81.9%		83.5%	82.0%	

^a^Study intake form allowed respondents to identify as transgender or other gender

Table 2 presents the total RSN and CSP costs and the average cost per member per month for clients who engaged with post-detox services. On average, RSN clients utilized services for a substantially longer time than CSP clients (4.5 versus 0.5 months). Some of this difference may be due to the case rate being active for 30 days from the last date of service. If we were to adjust the CSP time by adding 30 days to the last day of service, the average time in service would remain substantially different, 1.5 months for CSP clients versus 4.5 months for RSN clients. While the total cost per client over the study period was 53% higher for RSN clients ($1139) compared to CSP clients ($746), the average cost per member per month was significantly less ($255 versus $1458). To better understand this difference, we assessed service use patterns over time.

**Table 2:** Total RSN and CSP Costs Incurred Any Time After Index Detox Admission Based on Claims Data

	RSN	CSP
Number of Clients	816	924
Total amount Paid	$929,833	$689,578
Average Length of Stay (months)	4.5 months	0.5 months
Average Total Cost Per Member Per Month	$255	$1458
Average Total Cost Per Member	$1139	$746

**Fig. 1:**
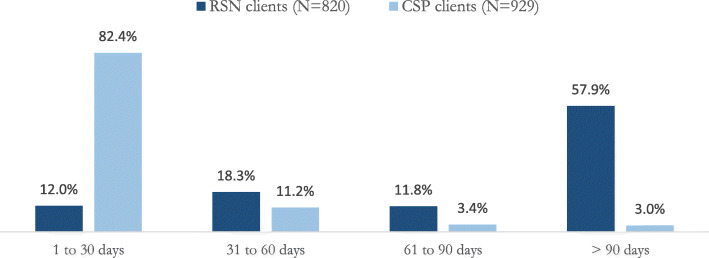
RSN and CSP length of engagement with service

Figure 1 presents the distribution of RSN and CSP service engagement by the proportion of clients that fell into different time frames based on 30-day increments. Contrasting patterns of engagement were observed. The overwhelming majority of CSP clients, 82.4%, utilized the service for 30 days or less. In contrast, over half (57.9%) of RSN clients utilized the service for more than 90 days and only 12.0% of clients utilized the service for 30 days or less.

Next, analyses focused on the type of RSN service provided, using RSN service data. Table 3 presents service contact and outreach information for 799 RSN clients. Service contacts could begin while the client was still at the detox and could be scheduled in advance, not requiring an outreach attempt for scheduling. Outreach attempts typically took place after a missed service attempt or after some time had passed without any type of regularly scheduled contact with the client. The data indicate that 97% of clients had a service contact after having enrolled. These clients averaged 6.5 contacts, with 64.6 min per contact on average.

**Table 3:** RSN Service Delivery Data on Contacts and Outreach Attempts (*N* = 799^a^)

	Service Contacts (excludes outreach)		Outreach Attempts (excludes service)	
	N	%	N	%
Clients with Service Contacts/Outreach Attempt	772	97%	362	45%
Number of Service Contacts/Outreach Attempts	5021		2290	
Average Number of Contacts/Attempts per Member	6.5		6.3	
**Time Spent:**	**Minutes**	**Hours**	**Minutes**	**Hours**
Total Time Spent	324,286	5404.8	11,612	193.5
Average Time per Contact/Outreach Attempt	64.6	1.1	5.1	0.08
Average Contact/Outreach Time per Member	420.1	7.0	32.1	0.53

^a^RSN service data were not available for 17 RSN clients

Nearly half (45%) of RSN clients had an outreach attempt, with an average of 6.3 attempts per client (*n* = 2290). RSNs spent a total of 193.5 h conducting outreach, with the average outreach attempt lasting approximately 5 min. RSNs spent approximately 32 min on outreach for each client they attempted to contact. Among the 799 RSN clients, 3.4% received outreach only, 54.7% received service contacts only, and 41.9% received both outreach and service contacts (Table 4).

**Table 4:** RSN Service: Types of Contacts for RSN Clients

Contact Type	N	%
Outreach Only	27	3.4%
Service Only^a^	437	54.7%
Both Outreach and Service	335	41.9%
TOTAL	799^b^	

^a^Service only contacts indicate contacts that did not require an outreach attempt for the service to take place

^b^RSN service data were not available for 17 RSN clients

### Qualitative findings on the role and impact of provider level context

**Fig. 2:**
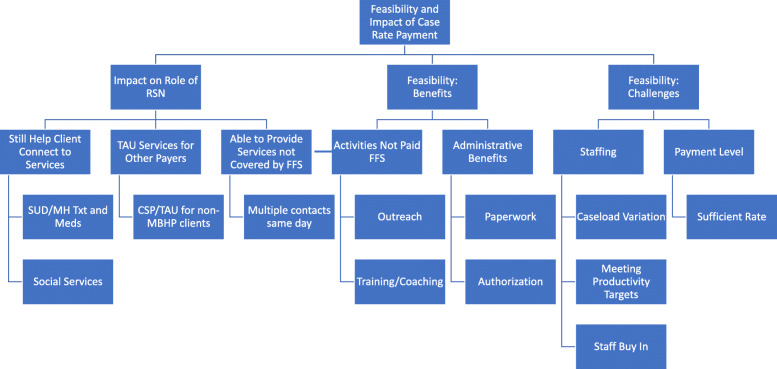
Coding structure for case rate qualitative interviews

Findings from the key informant interviews and LCs fell into three major themes: impact on RSN Role, feasibility: benefits, and feasibility: challenges, and were consistent across sites. Within these themes, sub-themes emerged related to services provided to clients, training and coaching, prior authorization, staffing and payment level (see Fig. 2). All themes arising from the data are reported below.

Respondents indicated that RSN and CSP staff shared a commitment to help clients engage in further treatment and believed that the detox facility was the ideal place to recruit clients for their service. Focusing on the context and experience of RSNs at the intervention sites, the shift to case rate billing represented a substantial change in procedures and processes. As such, buy-in among workers and their supervisors was crucial.

Within intervention sites, there was an early unexpected resistance to the case rate, which was originally set at $8.40 per day. Though later debunked, many RSNs perceived this as the equivalent of being paid $1 per hour. RSNs also struggled to reconcile productivity targets already established under the unit-of-service system with the new case rate system. This created fears that they might not be able to meet performance expectations, which could cost them their jobs.

Further, respondents noted that because providers serve clients with different insurance plans, and only MBHP adopted the new RSN service, many RSNs continued to provide CSP services to non-MBHP clients (i.e., clients not in the study), curtailing some of the intended benefits of the case rate (e.g., simplification of paperwork, no need to request authorizations). We were not able to measure how much CSP-related work with non-study clients impinged on RSN-related tasks, nor how CSP workload and related productivity targets impacted RSNs’ ability to recruit new clients or continue work with existing clients. However, RSNs were frequently challenged by having to navigate both payment systems simultaneously, even as they became more comfortable with the case rate over time.

RSN staffing levels represented a constant challenge. At the outset of the study, providers staffed the RSN positions based on their experience staffing for CSP positions, with no formal cap on caseload size. Over the course of the study, providers varied widely in terms of the caseload each RSN was expected to carry. This variation impacted the number of RSNs hired and the ability of RSNs to provide care to their clients. Additionally, providers hesitated to add new RSNs until they were certain new staff would have an appropriate minimum caseload. This resulted in caseload fluctuations throughout the project. Although the number of CSP staff also varied over time, this was less of an issue for providers that paid staff FFS.

Despite these challenges, positive impacts of the case rate were reported. When discussing the impact of case rate billing on service delivery, RSNs and their supervisors frequently highlighted how the case rate increased RSNs’ ability to access group trainings or coaching activities that they would have struggled to attend under the FFS system. Moreover, RSNs valued the unfettered ability to conduct outreach to connect with clients who might be struggling with sustaining motivation for treatment. RSNs felt this level of outreach helped them retain clients in services longer, as they could reconnect with clients who had not been in contact for a while. Finally, they appreciated the reduction in administrative burden resulting from the case rate payment method, compared to billing FFS.

## Discussion

Using the MRC framework to assess feasibility of case rate billing, we focused on whether case rate billing offered an economical alternative to FFS billing, whether the case rate payment method improved client length of engagement for clients receiving RSN services compared to clients receiving CSP services paid FFS, and how advantages and disadvantages of the case rate payment method influenced provider practices. Another consideration is whether and how the case rate advanced or supported the CMS’ triple aims of healthcare: reducing per capita costs, improving population health (increasing access), and improving the individual’s experience of care (quality) [24].

Our analysis indicates that the total cost of RSN services paid via the case rate was approximately 50% higher than for clients using CSP services paid FFS. At first blush, it appears the case rate failed the first test of feasibility in terms of offering an economical alternative to FFS billing. However, the answer is much more nuanced. RSN clients engaged with the service over a significantly longer period of time, resulting in a lower per member per month cost. Further, longer engagement has been shown to impact the quantity and quality of community-based services utilized by clients [25]. Analysis of the full claims data sample assessing service use and health care costs supports this assertion, showing increased rates of initiation with SUD treatment [26] and a shift in service use from more to less acute settings for the intervention group sample compared to TAU [18]. It also showed a slower rate of growth in health care spending for intervention group members than for TAU [18].

In terms of provider experience, simplicity of billing under a case rate payment model was an advantage. Case rate billing relieves administrative burden for providers, a benefit that also extends to payers. However, organizations and staff can only fully realize the administrative benefits of case rate payment if it is adopted by all or most payers. As noted earlier, the case rate improves engagement within the service by allowing for outreach not covered under FFS billing, and improves quality by creating opportunities for RSNs to participate in on-going coaching and training activities without consequence to their income or productivity targets.

Finally, staff faced challenges implementing the case rate approach. First, staff had to re-conceptualize how they used their time and monitored productivity. Second, staffing levels for RSNs caseloads fluctuated throughout the study. If caseload counts were very high or if they included very complex clients that required more time, clients may have had to wait longer for services, reducing access and possibly artificially extending the duration of service. Yet, any such delay would be limited by the case rate requirement that RSNs have contact with clients at least once every 30 days.

Supporting the argument for feasibility, case rate billing supported longer engagement with services, which can increase access to needed care while also improving overall health [27]. The case rate also facilitated RSN investment in training, coaching and education, supporting higher quality care. Of greatest importance, longer engagement and increased connections to community-based services produces cost savings across systems of care by connecting individuals with appropriate community-based care and reducing inappropriate or costly emergency room visits [18]. However, it is unclear how a payer, like MBHP, would be able to recoup any savings that occurred outside behavioral health.

At the conclusion of the study, MBHP continued implementing case rate billing for RSN services. Effective July 2018, MassHealth added RSN services as a covered benefit, including MBHP members, but it chose to pay using unit rates rather than case rates. This study demonstrates that case rates offer an alternative payment approach that can benefit both providers and consumers. However, until we see broader adoption across payers, providers may not realize the full benefits of case rate payments.

### Limitations

Despite our large sample size and the depth of the data available for analysis, the study had some limitations. This intervention contained many interacting components and changes in the payment approach may have coincided with other changes, thereby influencing our findings. For example, the trainings administered to RSN workers could have impacted practice patterns and RSNs’ abilities to engage with clients. Therefore, some of the changes observed may result from training and not just the case rate. On the other hand, the case rate payment structure made it easier for RSNs to fully participate in the trainings.

This study built on an existing workforce and service. As such, variation in the implementation, utilization and management of RSN or CSP services across providers existed before the study and the study did not require them to make any changes to participate. It is unknown whether or how these variations impacted uptake of either service. The intervention was at the site level and study subjects’ treatment assignments were not randomized, reducing potential for causal inferences. Lastly, the quantitative analysis did not control for differences across sites nor group assignment in the analysis. We did not control for these differences because we were not running predictive models. We were assessing the feasibility of the case rate and, thus, looking at actual units billed and paid for under the case rate. However, we recognize that site and group differences could have impacted the results.

## Conclusion

Assessing the *overall* contribution of the case rate in relation to outcomes we found that case rate billing could be a feasible alternative to FFS billing for RSN/CSP services for this population; although there are important caveats to consider.

Our findings highlight the ways in which case rate payment provides value and facilitates successful client engagement. However, when we focus on the provider-level context, issues based on the variation in payment model for RSN versus CSP services rise to the surface. Most providers work with multiple payers. If only one payer offers this model of payment for a widely used service, any accrued benefits may be minimized or lost.

Despite growing emphasis on healthcare payment reform, which involves episode, global or other capitated payments from payers to healthcare systems [28, 29], payments from healthcare systems to providers typically remain FFS. If adopted broadly, case rates may offer an opportunity to increase client engagement and quality of care while reducing overall healthcare costs.

## Supplementary Information

**Additional file 1: Table S1a.** Characteristics of Larger Study Sample by Programs – Full Claims Data Sample (*N*=4,491). **Table S1b.** Characteristics of Clients Who Utilized RSN or CSP Services by Programs – Subset of clients who utilized RSN or CSP Services (*N*=1,740).

## Data Availability

The data and materials used for this sub-study are from the state Medicaid provider and are not publicly available.

## Abbreviations

CMS: Centers for Medicare and Medicaid Services
CSP: Community Support Program staff
FFS: Fee-for-Service billing
ICD-9: International Classification of Diseases, Ninth Revision
LC: Learning Collaborative
MBHP: Massachusetts Behavioral Health Partnership
MRC: Medical Research Council
PCC: Primary Care Clinician
RSN: Recovery Support Navigator
SUD: Substance Use Disorder
TAU: Treatment-as-Usual
