# Supplementary material for: Evaluating the feasibility and impact of case rate payment for recovery support navigator services: a mixed methods study
Source: BMC Health Serv Res. 2020 Nov 3;20:1004. doi: 10.1186/s12913-020-05861-8 (PMC7607694; doi:10.1186/s12913-020-05861-8)
Supplement: Supplementary file 1 — Additional file 1: Table S1a. Characteristics of Larger Study Sample by Programs – Full Claims Data Sample (N=4,491). Table S1b. Characteristics of Clients Who Utilized RSN or CSP Services by Programs – Subset of clients who utilized RSN or CSP Services (N=1,740). [file 12913_2020_5861_MOESM1_ESM.docx]

**Table 1a. Characteristics of Larger Study Sample by Programs – Full Claims Data Sample (N=4,491)**

|  | **Intervention** | **TAU** | ***p-value*** | **P1** | **P2** | **P3** | **P4** | **P5** | **P6** | **P7** | **P8** | **P9** | **P10** | **P11** | **P12** | **P13** | **P14** | **P15** | **P16** | ***p-value*** |
| --- | --- | --- | --- | --- | --- | --- | --- | --- | --- | --- | --- | --- | --- | --- | --- | --- | --- | --- | --- | --- |
| Number of clients | 2667 | 1824 |  | 109 | 203 | 254 | 124 | 294 | 594 | 164 | 698 | 227 | 137 | 350 | 102 | 248 | 78 | 456 | 453 |  |
| Gender^a^ | % | % | 0.00 | % | % | % | % | % | % | % | % | % | % | % | % | % | % | % | % | 0.00 |
| Female | 34.8 | 28.6 |  | 0.0 | 35.0 | 25.6 | 100.0 | 32.0 | 36.4 | 31.1 | 34.0 | 30.8 | 26.3 | 31.4 | 38.2 | 23.4 | 23.1 | 26.1 | 31.4 |  |
| Male | 65.2 | 71.4 |  | 100.0 | 65.0 | 74.4 | 0.0 | 68.0 | 63.6 | 68.9 | 66.0 | 69.2 | 73.7 | 68.6 | 61.8 | 76.6 | 76.9 | 73.9 | 68.6 |  |
| Age |  |  | 0.00 |  |  |  |  |  |  |  |  |  |  |  |  |  |  |  |  | 0.00 |
| 18-24 | 13.7 | 8.4 |  | 16.5 | 12.3 | 14.2 | 27.4 | 19.7 | 15.3 | 14.0 | 6.2 | 16.3 | 8.8 | 5.2 | 16.7 | 7.3 | 14.1 | 7.2 | 9.7 |  |
| 25-29 | 20.5 | 15.8 |  | 22.9 | 18.2 | 27.9 | 24.2 | 24.5 | 22.4 | 15.9 | 13.9 | 24.2 | 10.9 | 12.3 | 16.7 | 11.3 | 14.1 | 18.6 | 19.7 |  |
| 30-39 | 30.2 | 30.3 |  | 22.9 | 33.0 | 30.3 | 23.4 | 31.3 | 31.8 | 33.5 | 28.8 | 31.7 | 34.3 | 31.4 | 21.5 | 27.8 | 24.4 | 30.3 | 32.4 |  |
| 40+ | 35.6 | 45.5 |  | 37.7 | 36.5 | 27.6 | 25.0 | 24.5 | 30.5 | 36.6 | 51.1 | 27.8 | 46.0 | 51.1 | 45.1 | 53.6 | 47.4 | 43.9 | 38.2 |  |
| Prior detox past year |  |  |  |  |  |  |  |  |  |  |  |  |  |  |  |  |  |  |  |  |
| ≤ 90 days | 51.6 | 53.0 | 0.35 | 50.5 | 52.2 | 44.9 | 56.4 | 52.7 | 53.4 | 47.0 | 52.6 | 51.6 | 54.7 | 54.0 | 44.1 | 44.8 | 51.3 | 52.6 | 58.9 | 0.04 |
| Charlson Comorbidity Score |  |  | 0.00 |  |  |  |  |  |  |  |  |  |  |  |  |  |  |  |  | 0.00 |
| 0 | 59.1 | 51.5 |  | 54.1 | 55.2 | 59.8 | 62.1 | 65.3 | 66.3 | 65.2 | 48.4 | 63.5 | 51.1 | 46.0 | 58.8 | 42.3 | 57.7 | 51.5 | 58.3 |  |
| 1 | 25.0 | 25.7 |  | 28.5 | 25.6 | 29.1 | 19.3 | 24.5 | 22.4 | 23.2 | 27.8 | 22.0 | 32.9 | 26.6 | 26.5 | 31.5 | 18.0 | 27.0 | 19.6 |  |
| 2 | 7.9 | 9.9 |  | 10.1 | 8.4 | 7.1 | 7.3 | 3.7 | 6.9 | 7.3 | 10.5 | 8.8 | 10.2 | 8.9 | 7.8 | 12.9 | 7.7 | 11.2 | 8.4 |  |
| 3 | 2.6 | 3.7 |  | 1.8 | 5.4 | 2.4 | 4.0 | 2.4 | 1.4 | 1.2 | 3.1 | 2.2 | 0.7 | 3.1 | 3.0 | 3.6 | 12.8 | 2.6 | 4.6 |  |
| 4+ | 5.4 | 9.2 |  | 5.5 | 5.4 | 1.6 | 7.3 | 4.1 | 3.0 | 3.1 | 10.2 | 3.5 | 5.1 | 15.4 | 3.9 | 9.7 | 3.8 | 7.7 | 9.1 |  |
| At least 1 Mental health diagnosis | 77.8 | 79.0 | 0.34 | 83.5 | 78.3 | 74.4 | 85.5 | 68.4 | 79.8 | 74.4 | 80.2 | 76.2 | 69.3 | 76.9 | 78.4 | 83.5 | 80.8 | 81.4 | 78.6 | 0.00 |
| Medicaid Enrollment Category |  |  | 0.00 |  |  |  |  |  |  |  |  |  |  |  |  |  |  |  |  | 0.00 |
| Disabled | 29.0 | 38.1 |  | 33.0 | 37.6 | 21.0 | 34.2 | 18.4 | 23.9 | 27.6 | 40.2 | 18.9 | 39.9 | 43.3 | 25.5 | 52.0 | 36.3 | 37.2 | 30.0 |  |
| Non-disabled (TANF) | 22.7 | 17.5 |  | 13.8 | 22.8 | 21.0 | 44.7 | 30.2 | 24.3 | 22.7 | 16.4 | 22.5 | 18.8 | 17.6 | 31.4 | 11.7 | 20.8 | 16.7 | 17.1 |  |
| Basic | 8.3 | 10.0 |  | 5.5 | 4.0 | 12.6 | 1.6 | 8.0 | 5.4 | 18.4 | 8.1 | 14.0 | 10.5 | 8.7 | 13.7 | 9.7 | 13.0 | 10.4 | 9.6 |  |
| Essential | 40.0 | 34.4 |  | 47.7 | 35.6 | 45.4 | 19.5 | 43.4 | 46.4 | 31.3 | 35.3 | 44.6 | 30.8 | 30.4 | 29.4 | 26.6 | 29.9 | 35.7 | 43.3 |  |
| Medicaid enrolled prior to index detox |  |  | 0.53 |  |  |  |  |  |  |  |  |  |  |  |  |  |  |  |  | 0.05 |
| ≤ 1 year | 18.9 | 18.1 |  | 18.4 | 22.3 | 14.6 | 19.5 | 21.2 | 19.6 | 19.0 | 18.2 | 17.6 | 22.6 | 21.7 | 9.8 | 12.1 | 11.7 | 18.9 | 19.6 |  |
| > 1 year | 81.1 | 81.9 |  | 81.6 | 77.7 | 85.4 | 80.5 | 78.8 | 80.4 | 81.0 | 81.8 | 82.4 | 77.4 | 78.3 | 90.2 | 87.9 | 88.3 | 81.1 | 80.4 |  |

a Study intake form allowed respondents to identify as transgender or other gender.

**Table 1b. Characteristics of Clients Who Utilized RSN or CSP Services by Programs – Subset of clients who utilized RSN or CSP Services (N=1,740)**

|  | **RSN** | **CSP** | **p-value** | **P1** | **P2** | **P3** | **P4** | **P5** | **P6** | **P7** | **P8** | **P9** | **P10** | **P11** | **P12** | **P13** | **P14** | **P15** | **P16** | ***p-value*** |
| --- | --- | --- | --- | --- | --- | --- | --- | --- | --- | --- | --- | --- | --- | --- | --- | --- | --- | --- | --- | --- |
| Number of clients | 816 | 924 |  | 14 | 37 | 63 | 31 | 142 | 190 | 76 | 203 | 60 | 55 | 142 | 42 | 130 | 31 | 244 | 280 |  |
| Gender^a^ | % | % | 0.05 | % | % | % | % | % | % | % | % | % | % | % | % | % | % | % | % | 0.00 |
| Female | 35.5 | 31.1 |  | 0.0 | 37.8 | 22.2 | 100.0 | 33.1 | 30.5 | 32.9 | 36.0 | 46.7 | 25.4 | 31.7 | 40.5 | 25.4 | 16.1 | 28.3 | 37.1 |  |
| Male | 64.5 | 68.9 |  | 100.0 | 62.2 | 77.8 | 0.0 | 66.9 | 69.5 | 67.1 | 64.0 | 53.3 | 74.6 | 68.3 | 59.5 | 74.6 | 83.9 | 71.7 | 62.9 |  |
| Age |  |  | 0.00 |  |  |  |  |  |  |  |  |  |  |  |  |  |  |  |  | 0.00 |
| 18-24 | 14.6 | 7.7 |  | 28.6 | 10.8 | 9.5 | 29.0 | 20.4 | 18.4 | 11.9 | 5.4 | 20.0 | 5.5 | 2.8 | 9.5 | 8.5 | 9.7 | 8.2 | 9.3 |  |
| 25-29 | 18.4 | 16.5 |  | 14.3 | 8.2 | 28.6 | 16.1 | 21.8 | 17.9 | 17.1 | 12.3 | 31.7 | 7.3 | 16.2 | 16.7 | 11.5 | 19.3 | 19.3 | 18.2 |  |
| 30-39 | 30.5 | 31.2 |  | 21.4 | 40.5 | 25.4 | 35.5 | 34.5 | 29.0 | 35.5 | 26.6 | 31.7 | 43.6 | 31.7 | 30.9 | 23.8 | 25.8 | 28.7 | 34.6 |  |
| 40+ | 36.5 | 44.6 |  | 35.7 | 40.5 | 36.5 | 19.4 | 23.3 | 34.7 | 35.5 | 55.7 | 16.6 | 43.6 | 49.3 | 42.9 | 56.2 | 45.2 | 43.8 | 37.9 |  |
| Prior detox past year |  |  |  |  |  |  |  |  |  |  |  |  |  |  |  |  |  |  |  |  |
| ≤ 90 days | 51.5 | 56.4 | 0.03 | 35.7 | 48.6 | 42.9 | 71.0 | 52.8 | 58.4 | 39.5 | 50.2 | 45.0 | 61.8 | 60.6 | 50.0 | 48.5 | 45.2 | 54.9 | 60.4 | 0.01 |
| Charlson Comorbidity Score |  |  | 0.00 |  |  |  |  |  |  |  |  |  |  |  |  |  |  |  |  | 0.00 |
| 0 | 60.7 | 48.6 |  | 50.0 | 54.1 | 60.3 | 67.7 | 68.3 | 69.5 | 63.2 | 43.8 | 71.6 | 38.2 | 39.4 | 57.1 | 40.0 | 54.8 | 48.8 | 57.2 |  |
| 1 | 24.5 | 27.8 |  | 42.9 | 27.0 | 25.4 | 16.1 | 20.4 | 24.2 | 26.3 | 28.6 | 16.7 | 38.2 | 31.0 | 28.6 | 33.8 | 19.4 | 28.7 | 21.4 |  |
| 2 | 7.6 | 11.0 |  | 7.1 | 10.8 | 6.3 | 6.5 | 5.0 | 5.3 | 6.6 | 12.3 | 6.7 | 16.4 | 8.5 | 9.5 | 13.1 | 6.5 | 13.5 | 8.9 |  |
| 3 | 2.2 | 3.6 |  | 0.0 | 2.7 | 4.8 | 3.2 | 2.1 | 0.5 | 2.6 | 2.5 | 3.3 | 1.8 | 2.8 | 2.4 | 3.9 | 16.1 | 2.4 | 3.9 |  |
| 4+ | 5.0 | 9.0 |  | 0.0 | 5.4 | 3.2 | 6.5 | 4.2 | 0.5 | 1.3 | 12.8 | 1.7 | 5.4 | 18.3 | 2.4 | 9.2 | 3.2 | 6.6 | 8.6 |  |
| At least 1 Mental health diagnosis | 79.5 | 83.1 | 0.06 | 85.7 | 83.8 | 71.4 | 90.3 | 71.8 | 79.5 | 75.0 | 84.7 | 85.0 | 81.8 | 78.9 | 83.3 | 84.6 | 90.3 | 84.4 | 57.2 | 0.06 |
| Medicaid Enrollment Category |  |  | 0.00 |  |  |  |  |  |  |  |  |  |  |  |  |  |  |  |  | 0.00 |
| Disabled | 35.3 | 42.4 |  | 42.9 | 54.1 | 27.0 | 25.8 | 19.9 | 30.5 | 37.4 | 52.2 | 27.1 | 51.8 | 50.7 | 30.9 | 53.1 | 38.7 | 39.8 | 35.4 |  |
| Non-disabled (TANF) | 29.6 | 16.7 |  | 42.9 | 37.8 | 28.6 | 61.3 | 38.3 | 34.7 | 17.3 | 15.3 | 33.9 | 14.8 | 18.6 | 28.6 | 13.1 | 12.9 | 13.9 | 18.9 |  |
| Basic | 7.8 | 9.7 |  | 0.0 | 2.7 | 15.8 | 3.2 | 7.1 | 3.2 | 17.3 | 7.9 | 10.2 | 9.3 | 7.1 | 14.3 | 7.7 | 22.6 | 10.2 | 9.3 |  |
| Essential | 27.3 | 31.4 |  | 14.2 | 5.4 | 28.6 | 9.7 | 34.7 | 31.6 | 28.0 | 24.6 | 28.8 | 24.1 | 23.6 | 26.2 | 26.1 | 25.8 | 36.1 | 36.4 |  |
| Medicaid enrolled prior to index detox |  |  | 0.40 |  |  |  |  |  |  |  |  |  |  |  |  |  |  |  |  | 0.86 |
| ≤ 1 year | 16.5 | 18.0 |  | 14.3 | 16.2 | 15.9 | 16.1 | 19.9 | 17.4 | 14.7 | 14.8 | 15.2 | 24.1 | 20.7 | 9.5 | 13.1 | 16.1 | 18.4 | 18.9 |  |
| > 1 year | 83.5 | 82.0 |  | 85.7 | 83.8 | 84.1 | 83.9 | 80.1 | 82.6 | 85.3 | 85.2 | 84.8 | 75.9 | 79.3 | 90.5 | 86.9 | 83.9 | 81.6 | 81.1 |  |

a Study intake form allowed respondents to identify as transgender or other gender.
